# Finding and tracing human MSC in 3D microenvironments with the photoconvertible protein Dendra2

**DOI:** 10.1038/srep10079

**Published:** 2015-05-14

**Authors:** Hugo R. Caires, Maria Gomez-Lazaro, Carla M. Oliveira, David Gomes, Denisa D. Mateus, Carla Oliveira, Cristina C. Barrias, Mário A. Barbosa, Catarina R. Almeida

**Affiliations:** 1 Instituto de Investigação e Inovação em Saúde, Universidade do Porto, Portugal; 2 INEB - Instituto de Engenharia Biomédica, Universidade do Porto, Rua do Campo Alegre, 823, 4150-180 Porto, Portugal; 3 ICBAS - Instituto de Ciências Biomédicas Abel Salazar, Universidade do Porto, Rua Jorge Viterbo Ferreira, 228, 4050-313 Porto, Portugal; 4 b.IMAGE - Bioimaging Center for Biomaterials and Regenerative Therapies, INEB, Universidade do Porto, Rua do Campo Alegre 823, 4150-180 Porto, Portugal; 5 ISPUP - Instituto de Saúde Pública da Universidade do Porto, Rua das Taipas, 135, 4050-600 Porto, Portugal; 6 IPATIMUP - Instituto de Patologia e Imunologia Molecular da Universidade do Porto, Rua Dr. Roberto Frias s/n, 4200-465 Porto, Portugal; 7 Medical Faculty of the University of Porto, Alameda Hernani Monteiro, 4200-319 Porto, Portugal

## Abstract

Mesenchymal Stem/Stromal Cells (MSC) are a promising cell type for cell-based therapies - from tissue regeneration to treatment of autoimmune diseases - due to their capacity to migrate to damaged tissues, to differentiate in different lineages and to their immunomodulatory and paracrine properties. Here, a simple and reliable imaging technique was developed to study MSC dynamical behavior in natural and bioengineered 3D matrices. Human MSC were transfected to express a fluorescent photoswitchable protein, Dendra2, which was used to highlight and follow the same group of cells for more than seven days, even if removed from the microscope to the incubator. This strategy provided reliable tracking in 3D microenvironments with different properties, including the hydrogels Matrigel and alginate as well as chitosan porous scaffolds. Comparison of cells mobility within matrices with tuned physicochemical properties revealed that MSC embedded in Matrigel migrated 64% more with 5.2 mg protein/mL than with 9.6 mg/mL and that MSC embedded in RGD-alginate migrated 51% faster with 1% polymer concentration than in 2% RGD-alginate. This platform thus provides a straightforward approach to characterize MSC dynamics in 3D and has applications in the field of stem cell biology and for the development of biomaterials for tissue regeneration.

MSC are an attractive cell source for regenerative cell-based therapies due to its well established multipotency, immunomodulatory and paracrine properties, combined with their ability to migrate into damaged tissues. Most strategies being currently explored involve either stem cell transplantation to patients^1^, implantation of the triad scaffolds/stem cells/growth factors^3^, or the use of materials that stimulate endogenous stem cell recruitment^4^. Regardless of the approach, to improve the effectiveness of such applications it is critical to understand the determinants of stem cell migration in 3D microenvironments.

Despite the worldwide effort to modulate and direct stem cell fate for tissue regeneration, the influence of 3D extracellular matrix (ECM) features on MSC motility still remains largely unknown. A tool to longitudinally follow MSC in 3D is essential to better understand cell migration and organization in 3D microenvironments. To this end, a number of cell labeling techniques have been developed. Some studies have suggested that cell transfection with fluorescent proteins^7^ will be required for long term cell tracking and others are pursuing magnetic nanoparticles^8^, bioluminescent probes^1^, quantum dots^3^ and radioactive isotope alternatives^4^ for stem cell labeling.

Most of these bioimaging techniques are well established but are more oriented to localize the final homing site of transplanted cells or to roughly quantify cell survival after implantation, being rather limited for studying the effect of cell-ECM interactions on MSC dynamical behavior. There are some reports on the use of fluorescent proteins or dyes to track stem cells, but most use labeling in order to find the cells at one fixed time point and not to follow them by time-lapse analysis^5^. As MSC are slowly moving cells when in 3D, tracing the same cells through time requires imaging performed for several days and it is usually not feasible to keep the cells under the microscope for long periods. Apart from unpractical to perform continuous 3D live cell imaging for more than a day, the increased probability of photo-damaging cells or photobleach the fluorophore over time is often the limiting factor. When imaging cells at discrete daily intervals, it is extremely difficult to find exactly the same cells that were being imaged^6^. Thus, studies aiming to compare the dynamic MSC behavior within distinct 3D microenvironments would benefit from new bioimaging tools that allow finding and tracing the same cells over long periods of time.

A class of fluorescent proteins that can change irreversibly the emission fluorescence spectrum upon laser excitation offers a promising and flexible opportunity for solving this problem^8^. With these fluorescent photoconvertible proteins, it is possible to create a reference by selecting and photo-marking cells of interest in precisely defined positions, which will then be easier to track with no mistaken identity issues. It has been shown that photoswitchable or photoconvertible proteins can indeed be applied to accurately track a discrete sub-population of tumor cells even with imaging sessions spaced over days^0^. Dendra2 is such a photoswitchable protein, with a fluorescence spectrum similar to Green Fluorescent Protein (GFP), but that upon exposure to blue light (e.g., 405 nm) suffers an irreversible shift to red (>150 nm). Dendra2 is highly photostable after switching to its red form, and some of the advantages compared with other photoswitchable proteins are: its predominant monomeric form (generating less aggregates than tetrameric proteins); the fact that it forms a completely functional chromophore at 37 °C, and its great brightness in relation to other photoconvertible proteins^2^.

Here, Dendra2+ human bone marrow MSC were generated and characterized, and the influence of optimized photoconversion on MSC properties was analyzed. Finally, the feasibility and flexibility of Dendra2 imaging to quantify MSC dynamics in distinct 3D environments was verified. A tool to investigate and compare the dynamical behavior of MSC in bioengineered materials was thus developed.

## Results

### Transfection of human MSC to express the photoswitchable protein Dendra2

**Figure 1:**
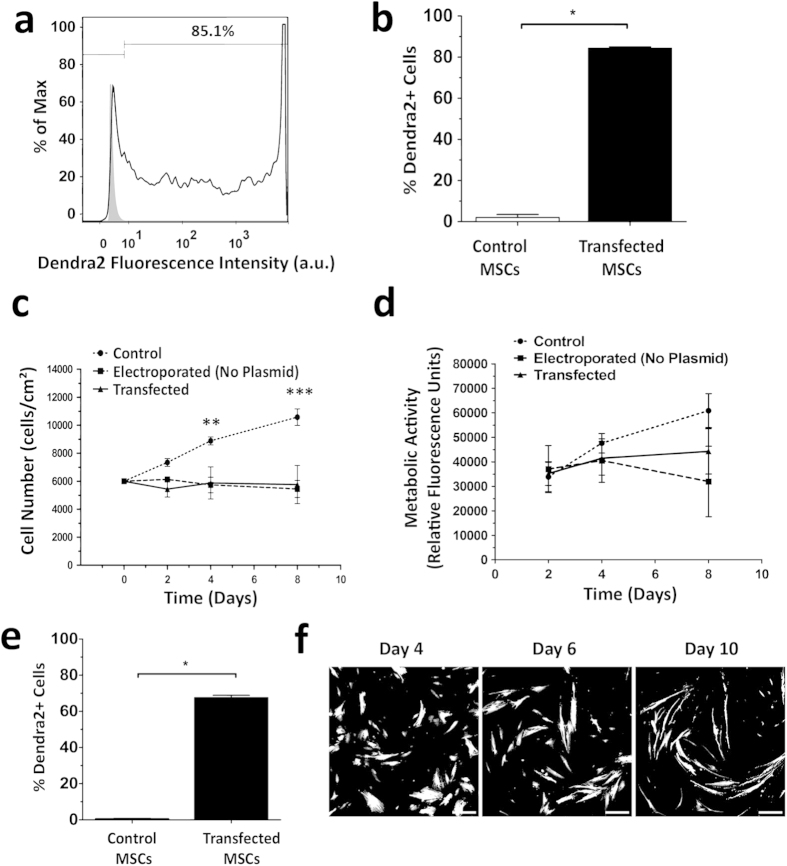
Transfection of human MSC to express Dendra2. (**a**) Flow cytometry histogram showing the fluorescence intensity of control (grey, filled) and transfected (open) MSC two days after electroporation. Data is representative of 3 experiments. (**b**) The percentage of Dendra2+ MSC upon electroporation was measured by flow cytometry. Graphs show mean ± SEM of three experiments. ^*^p > 0.05 by Mann-Whitney test. (**c**) Growth curve of MSC over a period of 8 days after an initial seeding of 6,000 cells/cm^2^. Number of cells was determined by trypan blue exclusion. Graph shows mean ± SEM of three experiments. ^**^p > 0.01 and, ^***^p > 0.001 by two-way ANOVA with Bonferroni post-test for comparison with control group. (**d**) Relative fluorescence unit (RFU) as a measure of metabolic activity determined by resazurin assays. Graph shows mean ± SEM of four experiments. Differences are not statistically significant, two-way ANOVA with Bonferroni post-test for comparison with control group. (**e**) The percentage of Dendra2+ cells was determined 10 days after transfection by flow cytometry. Graphs show mean ± SEM of three experiments. ^*^p > 0.05 by Mann-Whitney test. (**f**) Fluorescence microscopy images of transfected cells 4, 6 and 10 days after electroporation. Scale bar, 250 μm.

To follow living MSC in 3D for long periods of time it is vital to develop a strategy that allows imaging of the same cells at different time-points. Here, human bone marrow MSC were transfected to express a photoswitchable fluorescent protein, Dendra2, which enables green-to-red photo-conversion of labeled MSC, and subsequently highlighting and tracking the same group of cells over long time periods. Transfection of MSC by electroporation yielded more than 80% of cells expressing Dendra2 protein, with most cells expressing high levels of this fluorescent protein (Fig. 1a,b). Transfection with the pDendra2 plasmid did not affect cell number or metabolic activity of MSC, which remained constant for up to eight days, when comparing with cells electroporated in the absence of a plasmid. However, electroporation in itself affected the kinetics of the cell number (Fig. 1c,d). Importantly, almost 70% of the transfected cell population still expressed Dendra2 ten days after transfection (Fig. 1e,f). Additionally, MSC morphology was not affected by transfection; transfected MSC started to exhibit an elongated spindle shape after ten days due to extended cell culture time (Fig. 1f).

### Characterization of Dendra2+ MSC

**Figure 2:**
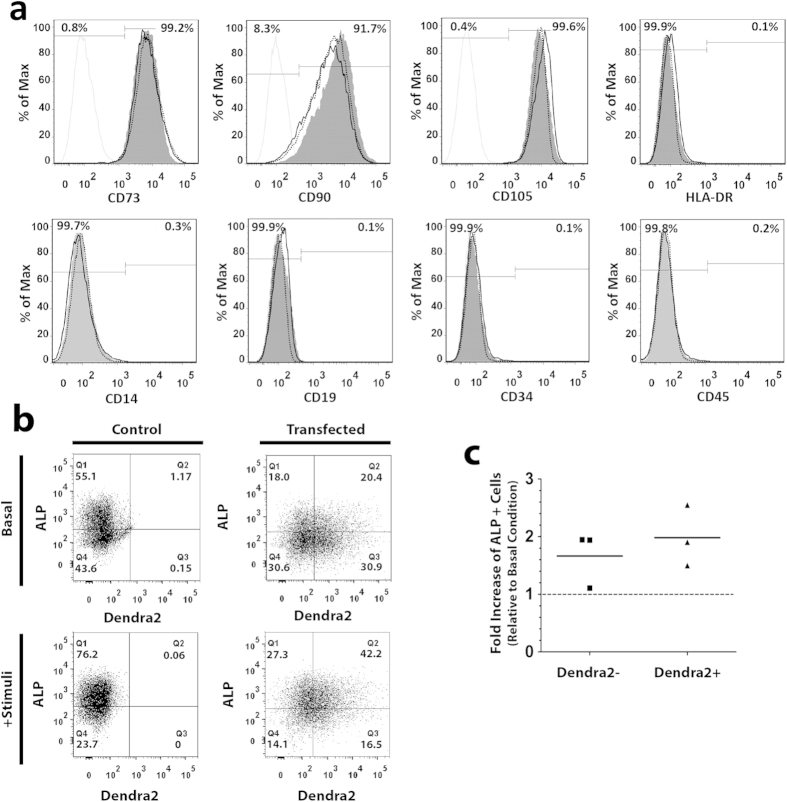
Dendra2 expression does not interfere with MSC properties. (**a**) Flow cytometry histograms showing expression of CD14, CD19, CD34, CD45, HLA-DR, CD73, CD90 and CD105 by control (grey filled) and transfected MSC (Dendra2+, solid line; Dendra2−, dashed line). Isotype control is displayed as the grey line. Numbers indicate the percentage of cells in each gate for the Dendra2+ cells. Figure is representative of three experiments. (**b**) Representative flow cytometry dot plots showing ALP surface staining on control or transfected MSC cultured under basal or osteogenic medium for 7 days (n = 3). (**c**) Fold increase of the percentage of ALP+ cells in Dendra2+ and Dendra2− MSC cultured in osteogenic conditions for 7 days, in relation to cells cultured in basal medium (n = 3). Each dot represents data from one experiment and bars represent the mean. Differences are not statistically significant, Friedman test with Dunns post-test comparison.

In order to analyze whether Dendra2 expression affected MSC properties, we firstly analyzed the expression profile of MSC-related cell surface antigens. MSC were electroporated to obtain a mixed population with Dendra2 positive and negative cells, to correlate with expression of the surface markers. Importantly, neither electroporation nor Dendra2 expression influenced surface marker expression, with more than 90–95% of cells being positive for CD73, CD90 and CD105 and negative for CD14, CD19, CD34, CD45, HLA-DR (Fig. 2a).

To test whether Dendra2 transfection affects MSC differentiation capacity, control and transfected cells were induced into osteogenic and adipogenic differentiation. Both transfected and control cells were able to differentiate when cultured under osteogenic or adipogenic stimuli but not under basal condition, as detected by Alkaline Phosphatase activity (ALP), von Kossa and oil red O stainings (Supplementary Fig. 1a,b). Furthermore, both Dendra2+ and Dendra2− MSC could be visualized by fluorescence microscopy to express ALP and stain with oil red O under the appropriate stimuli (Supplementary Fig. 1c). Moreover, the percentage of ALP+ MSC increased after culture with osteogenic stimuli for 7 days both on Dendra2+ and Dendra2− cells (Fig. 2b,c). Thus, Dendra2 expression did not interfere with either MSC surface marker expression nor MSC differentiation capacity.

### Efficient Photoswitching of Dendra2 protein expressed by MSC

**Figure 3:**
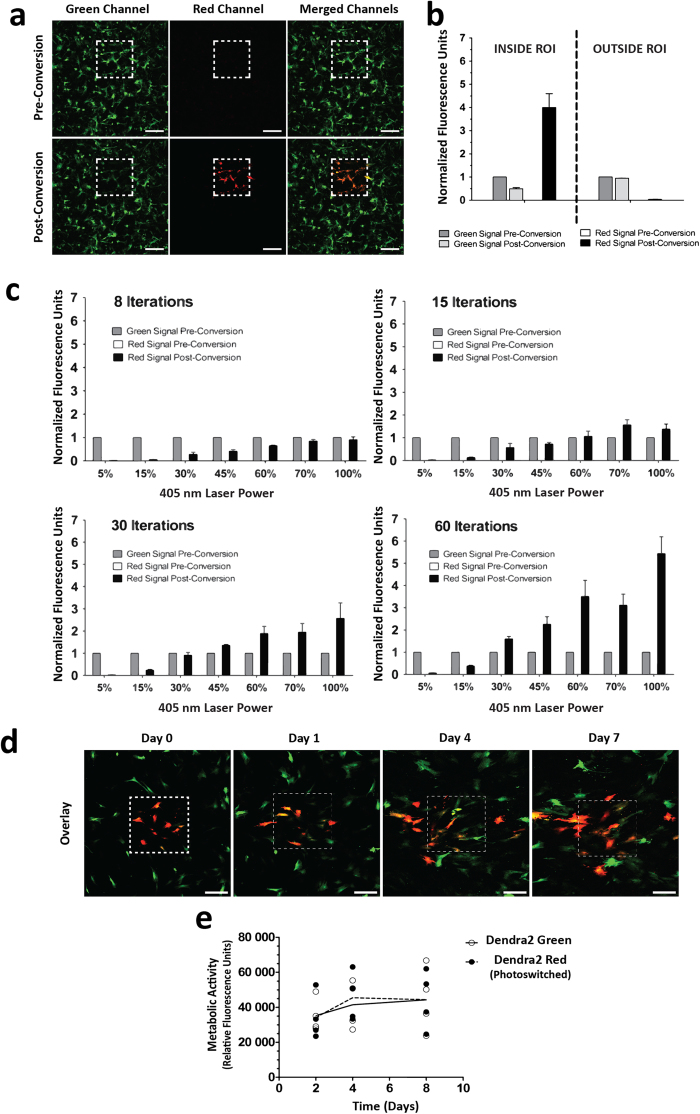
Photoswitching Dendra2 protein expressed by MSC. (**a**) Confocal microscopy images in green, red and merged channels of Dendra2+ MSC taken before (top) and after (bottom) irradiation with a 405 nm laser. Photoconversion was achieved by laser irradiation of a square region of interest (ROI) targeting a selected cell population (dashed line). Scale bar, 250 μm. (**b**) Green-to-red conversion did not induce photoconversion of cells located outside the defined ROI. Fluorescence intensity in each channel was measured and normalized relatively to the fluorescence intensity in the green channel before conversion. (**c**) The laser power and number of iterations affected photoconversion efficiency of Dendra2 on MSC. (**b**) and (**c**) Mean ± SEM of normalized fluorescence units (n = 3 experiments, 3 cells analyzed in each). (**d**) Confocal microscopy images (merged green and red channels) of Dendra2 expressing MSC taken after irradiation. Green-to-red conversion for a selected group of cells was achieved at time-point zero and used to track this group of cells for 7 days after conversion. Scale bar, 250 μm. (**e**) Relative fluorescence units (RFU) as a measure of metabolic activity of Dendra2 Green and Dendra2 Red MSC, determined 2, 4 and 8 days after an initial seeding of 6,000 cells/cm^2^. Photoconversion was performed at day 2. Each dot represents data of one individual experiment, lines represent the mean. Differences are not statistically significant, two-way ANOVA with Bonferroni post-test.

Maximized photoconversion efficiency should result in enhanced signal-to-noise ratio of photo-marked MSC and therefore in improved imaging quality and extended tracking time. Excitation at 488 nm with high power could lead to photoconversion but using a 405 nm laser was much more efficient. Hence, parameters were optimized for efficient photoconversion with a 405 nm laser in a region of interest (ROI), without complete fluorescence bleaching and without photoconverting nearby cells (Fig. 3a,b). To determine the optimal conditions for Dendra2 green-to-red photoconversion, we incrementally increased laser power and tested different numbers of iterations (Fig. 3c). Laser power below 15% was insufficient for noticeable photoconversion. Efficient photoconversion was achieved using 60–70% of laser power with 60 iterations, which allowed obtaining an intense red signal to track MSC for an extended time (Fig. 3d). UV irradiation with these optimized settings did not affect cells metabolic activity, indicating that it was not cytotoxic (Fig. 3e). Photoconversion could also be achieved at the subcellular level (Supplementary Fig. 2).

### Migration of Dendra2+ MSC in 2D

**Figure 4:**
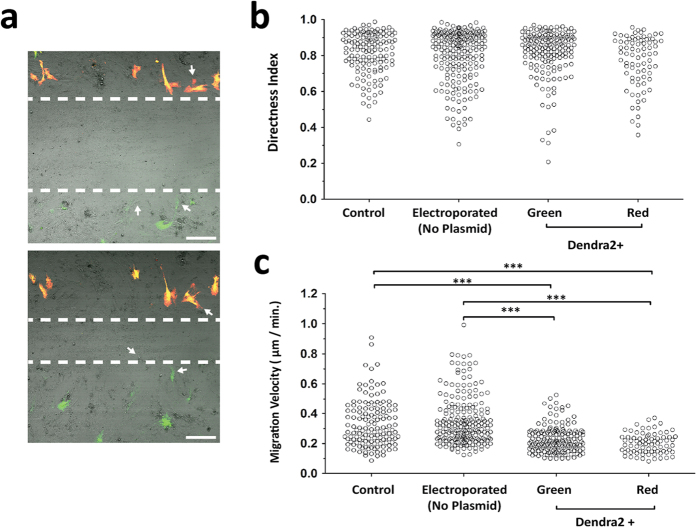
Migration of Dendra2+ MSC in 2D. (**a**) Representative images of *in vitro* wound healing time-lapse microscopy at time-points 0 (top) and 16.5 hours (bottom) post wound. Dashed lines represent the migrating front and arrows show Dendra2−, Dendra2+ Green and Dendra2+ Red cells. Scale bar, 250 μm. (**b**) Directness index and (**c**) migration velocity were determined for Control, Electroporated (without plasmid), Dendra2 Green and Red single cells (n = 4). Only cells initially at the migrating front were analyzed. ^***^p > 0.001, linear mixed model.

To examine the ability of Dendra2+ MSC to migrate, time-lapse microscopy of an *in vitro* wound healing assay was performed (Fig. 4). MSC migrated using lamellipodia mode with visible cell extensions and rear body retraction. Cells motility was oriented towards the center of the wound with a similar directness index for all groups (Fig. 4b). However, the velocity of MSC showed some differences: control cells migrated at 0.33 ± 0.15 μm/min, electroporated cells without pDNA at 0.35 ± 0.16 μm/min, transfected green cells at 0.23 ± 0.09 μm/min and photoswitched red cells at 0.20 ± 0.07 μm/min (Fig. 4c). Therefore, even though transfected cells moved slightly slower than control cells, photoswitching did not interfere with the cells velocity.

### Tracking Migration of MSC in different 3D matrices

**Figure 5:**
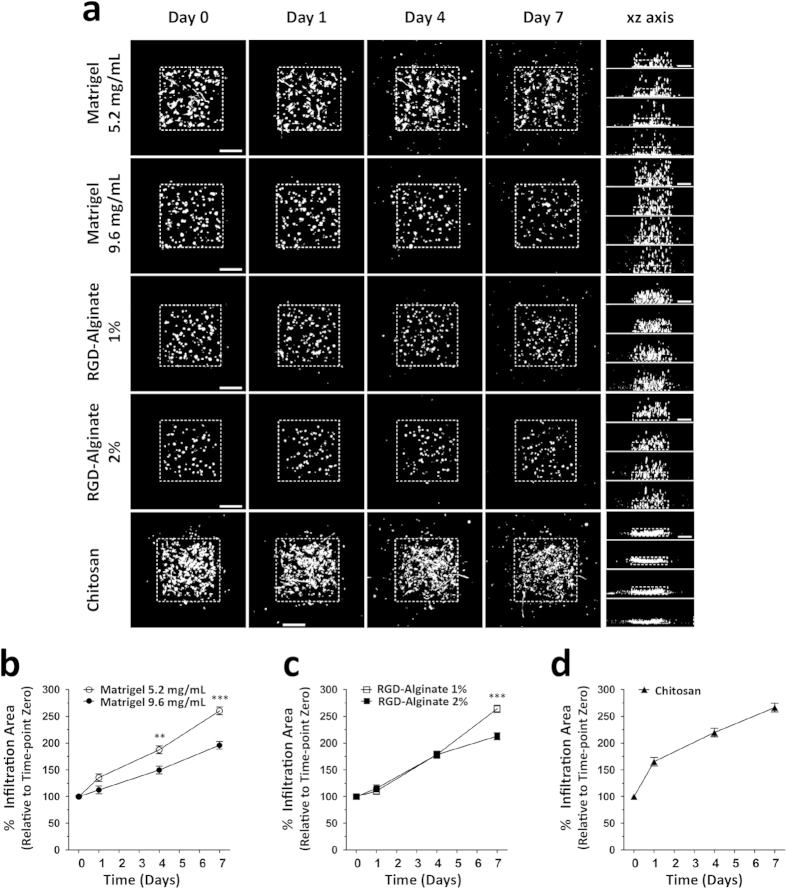
MSC motility in 3D microenvironments. (**a**) Representative maximum projection images of cells in Matrigel, RGD-alginate and Chitosan matrices obtained over a period of 7 days (first four columns in xy; right column in xz). Cell migration can be determined by the increasing infiltration area over time in maximum projection images. White dotted square line represents the photoconverted area at time-point zero. Scale bar, 250 μm. The infiltration area at time-points 1, 4 and 7 days was normalized to time-point zero, for cells in (**b**) Matrigel (5.2 or 9.6 mg/mL), (**c**) RGD-alginate (1% or 2%) hydrogels or in (**d**) Chitosan scaffolds. ^**^p > 0.01 and ^***^p > 0.001 by linear mixed model.

To assess the feasibility of this tool to analyze MSC dynamics in 3D, experiments were performed in microenvironments with distinct properties, including the hydrogels Matrigel and alginate as well as chitosan porous scaffolds. Transfected MSC were embedded in the hydrogels or seeded on top of chitosan scaffolds and tracked for up to seven days. It was possible to photoconvert cells, enabling finding and imaging the same cells at discrete daily intervals, after placing them in the 37 °C/CO_2_ incubator instead of leaving them for 7 days under the microscope. Thus it was possible to trace photoconverted cells over extended periods of cell culture, and to compare MSC motility in different matrices (Fig. 5). Labeling cells with a fluorescent protein such as GFP would not allow tracing the same cells over these long periods of time, as evident when cells were imaged only at the green channel (Supplementary Fig. 3). Approximately 400–500 μm in depth were effectively imaged for Matrigel and RGD-alginate hydrogels and approximately 100 μm for chitosan scaffolds due to lower light penetration properties of the later. To avoid any inaccuracy related with photoconversion in stiffer/opaque materials, which may derive from laser scattering, data was normalized to the photoconverted area at time-point zero. Unwanted photoconversion due to routine imaging with low power 488/561 nm lasers was not observed over the time points imaged.

**Figure 6:**
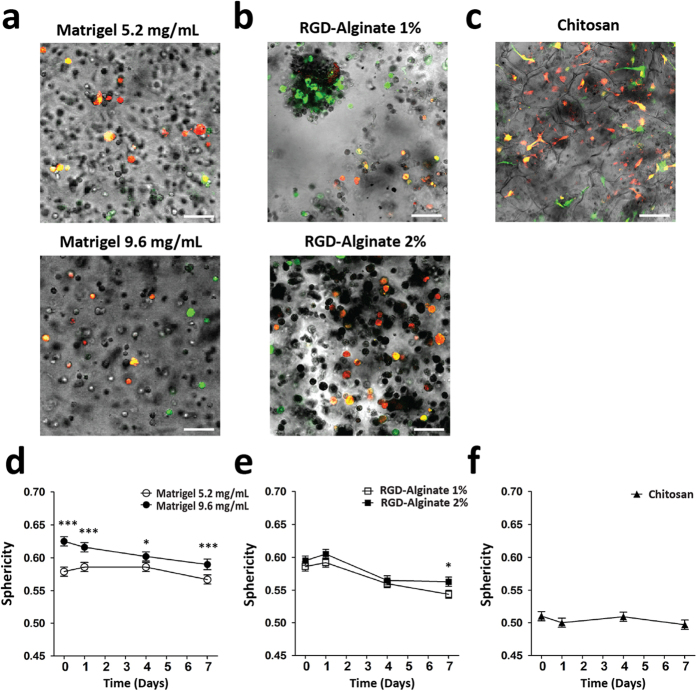
Dendra2+ MSC morphology in 3D microenvironments. Representative merged bright-field and fluorescence images of cells when embedded in (**a**) Matrigel (5.2 or 9.6 mg/mL), (**b**) RGD-alginate (1% or 2%) hydrogels or seeded in (**c**) Chitosan scaffolds at day eight. Scale bar, 100 μm. Cells sphericity for (**d**) Matrigel, (**e**) RGD-alginate and (**f**) Chitosan was determined throughout 7 days. Data are mean ± SEM of more than 150 cells per time point (3 experiments). ^*^p > 0.05 and ^***^p > 0.001 by linear mixed model.

This approach could be used to discriminate differences in MSC dynamics in 3D matrices with tuned physicochemical properties. Dendra2+ MSC were embedded in Matrigel with a protein content of 5.2 mg/mL and 9.6 mg/mL or in RGD-alginate at 1% and 2% (wt) polymer concentration. Then, a square region was photoswitched and the population was traced over a period of seven days. In Matrigel, MSC were able to migrate 64% more at 5.2 mg/mL than at 9.6 mg/mL (Fig. 5). Moreover, Matrigel 5.2 mg/mL favored a more elongated morphology than cells embedded in Matrigel 9.6 mg/mL (Fig. 6a,d). In RGD-alginate, MSC embedded in a 1% polymer concentration migrated 51% more than in 2% concentration after seven days of incubation (Fig. 5c), with more elongated and clustered cells in 1% RGD-alginate (Fig. 6b,e). Interestingly, cells seeded in the much different chitosan scaffolds showed an infiltration area similar to Matrigel 5.2 mg/mL and RGD-alginate 1% hydrogels at day 7, even though the increase in infiltration area was higher for early time points. MSC in chitosan scaffolds were the most elongated for all time-points (Fig. 6c,f). MSC embedded in Matrigel 9.6 mg/mL, 1% and 2% RGD-alginate but not in Matrigel 5.2 mg/mL or chitosan matrices displayed a statistically significant trend to decrease cell sphericity over time.

In summary, Dendra2 photoswitching in MSC allowed analysis of the cells morphology and quantification of MSC motility in different 3D microenvironments.

## Discussion

Here we describe an imaging tool to follow the dynamical behavior of human MSC in complex 3D microenvironments. The idea behind this method is to photo-mark Dendra2+ MSC in precisely defined positions, being then able to follow the same group of cells for long periods of time.

High transfection efficiency with minimal loss of Dendra2 expression after ten days was achieved by electroporation. Analysis of cell number suggests that there is equilibrium between proliferation and cell death in electroporated cells, an effect that was not affected by Dendra2 protein expression. Electroporation in itself (without any plasmid) affected the proliferation of these slowly dividing cells, whose number remained constant over time. However, the metabolic activity of the cells does not show statistically significant differences, indicating that the cells are alive and probably recovering from the electroporation. These data highlights the need that future studies involving Dendra2 transfection, which may be used to study the role of other proteins, also perform these controls. A diminished cell proliferation rate due to electroporation is a limitation of this technology, but at the same time it avoids the problem of signal loss due to fluorescence signal dilution to daughter cells which could limit cell tracking to a couple of days or less depending on the cells proliferation rate^4^.

Photo-marked red cells could be clearly distinguished from the green non-converted cells and exhibited a 3-fold increase in red fluorescence. This increase in red fluorescence intensity was coupled with a decrease in green fluorescence intensity, consistent with conversion of both the green and neutral forms of Dendra2 into the red form^5^. Photoswitching did not lead to diminished metabolic activity, suggesting that it was not cytotoxic. Also, cells could be tracked for at least seven days, indicating that the degradation rate of Dendra2 red form is low. Importantly, photoconversion only targeted cells inside a pre-defined ROI, with minimal photoconversion occurring outside the ROI. Photoswitching did not adversely affect MSC properties, nor the cells migration directionality and velocity. As transfection affected MSC motility, this tool does not allow quantification of the cells speed but it allows the comparison of the dynamic behaviour when cells are subjected to different treatments or microenvironments.

To analyze whether Dendra2 imaging allows detection of changes in MSC dynamics in 3D, we compared MSC migration in Matrigel, RGD-alginate hydrogels and in porous chitosan scaffolds. Matrigel is a reconstituted basement membrane widely used to study cellular invasion and interactions with the basement membrane. Cells embedded in diluted Matrigel 5.2 mg/mL migrated more than in Matrigel 9.6 mg/mL matrices. Also, cells in diluted Matrigel adopted a more elongated shape, which is usually associated with increased cell migration. The slower migration in denser Matrigel matrix could be explained by the tighter mesh size, the increased gel stiffness, the lower diffusion of soluble factors from culture medium and/or sub-optimal adhesion ligand density^6^. Alginate is a natural hydrogel forming polysaccharide that can be tailored with specific biofunctional moieties to modulate cell behavior and thus is currently being widely explored for tissue engineering^8^. A higher motility in 1% as compared with RGD-alginate 2% was seen after seven days of culture. Here, cell motility probably depends more on gel stiffness than on pore size, as cells are embedded and need to rearrange the mesh to create the physical space to move inside this matrix. Furthermore, mechano-sensing molecules can modulate cytoskeleton organization, which affects migration mode and speed^2^.

To further demonstrate the usefulness of this method in biomaterials where cells are seeded on top instead of embedded inside, MSC dynamical behavior was analyzed in porous chitosan scaffolds. Chitosan is a natural biodegradable polysaccharide obtained by N-deacetylation of chitin, currently being explored for many applications, from bone repair to gene therapy, amongst others. In chitosan scaffolds cells exhibited an elongated morphology through seven days of imaging possibly due to the high porosity and large pore size (larger pores and smaller interconnecting pores measure on average 147 μm and 58 μm in diameter^4^). On the other hand, the lack of physical constrain can impact negatively on cells migration velocity, as less adhesion sites could be available to support an efficient cell movement. Cells could be seen to migrate, with an infiltrated area similar to the values obtained for Matrigel 5.2 mg/mL and RGD-alginate 1% after seven days of incubation.

Applying this tool to access the behavior of the cells in different materials revealed some of the issues that may arise when dealing with matrices with varied optical properties. Indeed, the more opaque chitosan allowed imaging a smaller depth and led to some light scattering when photoswitching was performed. It was possible to overcome this by normalizing data to the photoconverted area at time-point zero. Table 1 compares some characteristics of the analysed scaffolds, as well as differences in the imaging strategies and the cellular behavior in the 3D matrices.

In summary, a tool that takes advantage of the photoswitchable Dendra2 was developed to characterize and compare MSC migration in distinct 3D microenvironments. Applying photoconvertible proteins has clear advantages to trace cells in 3D when comparing with fluorescent dyes or proteins such as GFP (Table 2). Importantly, this method allowed quantification of differences in cell migration in bioengineered matrices with fine-tuned physicochemical modifications. Furthermore, it also allowed coupling cell morphology analysis with quantification of cell migration in real-time, to study cell-ECM interactions. This method provides reliable tracking over at least seven days with an easy and straightforward analysis of cell migration using open source software. Thus, this novel platform to characterize MSC dynamics in matrices will likely have an important role in the development of biomaterials with improved characteristics for stem cell recruitment and migration or even for the study of stem cell biology in 3D microenvironments.

## Materials & Methods

### Cells

Human bone marrow MSC (purchased from Lonza) were cultured in MSC growth medium (DMEM with low glucose and Glutamax plus 10% selected inactivated FBS and 1% penicillin/streptomycin (all from Invitrogen)). Cells were incubated at 37 °C/5% (V/V) CO_2_ and medium was changed twice per week until cells reached approximately 80% confluence. For expansion, cells were detached by treatment with 0.05% trypsin/EDTA (Invitrogen) and replaced in 150 cm^2^ tissue culture flasks (BD Falcon). Cells were used at passages 6–10.

### MSC transfection

MSC were ressuspended in OptiMEM (Invitrogen) at 1.25 × 10^6^ cells/mL. Then, 200 μl of cell suspension were transferred to a 4 mm electroporation cuvette (BioRad), mixed with 60 μg Dendra2 plasmid DNA (pDendra2, Clontech) per 1 × 10^6^ cells and electroporated using a X-Cell Gene Pulser (BioRad). For optimal cell electroporation, an exponential pulse protocol was used with 250 V, 950 uF and 200 Ω^5^. After electroporation, cells were cultured in MSC growth medium at 37 °C/5%CO_2_. After 16–18 h, medium was changed to remove debris and dead cells. Cells were incubated for 48 h to allow Dendra2 expression. Electroporation was performed with 200 μl of cell suspension for all experiments except for surface marker expression and 2D motility analysis, where 500 μl were used to obtained an evenly proportion of Dendra2+ and Dendra2− cells.

To determine transfection efficiency, the percentage of Dendra2+ cells was determined 48 h after electroporation by flow cytometry (FACSCalibur, Becton Dickinson) and analysis with FlowJo software.

### Cell number, metabolic activity, and morphology

Cells were seeded in triplicate at 6,000 cells/cm^2^ in 24 well plates and metabolic activity was assessed as described^6^. To analyze the effects of photoconversion on cells metabolic activity, photoswitching was performed 2 days after transfection. To estimate cell number, three wells for each condition were analyzed by trypan blue exclusion. Morphology was visualized by confocal microscopy (Leica TCS SP5 II, Leica Microsystems, Wetzlar, Germany, model DMI6000B-CS).

### Surface marker expression

Cells were harvested 72 h after electroporating 500 μl cell suspension per cuvette. Surface staining was performed in 96-well plates by incubating for 30 min on ice with all antibodies diluted at 1.25:50, except for anti-CD73, at 2.5:50, in staining buffer (PBS, 0.5% BSA, 0.01% sodium azide), as determined after an initial titration. The antibodies used were: PerCP eFluor® 710 conjugated anti-human CD73 (clone AD2), APC conjugated anti-human CD90 (clone 5E10), eFluor® 450 conjugated anti-human CD45 (clone HI30), PE conjugated anti-human CD105 (clone SN6), eFluor® 450 conjugated anti-human HLA-DR (clone L243, eBioscience) and eFluor® 450 conjugated anti-human CD34 (clone 4H11), all from eBioscience, and PE conjugated anti-human CD14 (clone MEM-15), APC conjugated anti-human CD19 (clone LT19), from Immunotools. Appropriate isotype controls were used. Samples were washed four times in staining buffer before acquisition in a flow cytometer (FACSCanto II, Becton Dickinson). Data were analyzed with FlowJo software. Only samples resulting in more than 10,000 events were analyzed.

### Measuring ALP expression by flow cytometry

MSC were plated at 30,000 cells/well in 24 well plates with basal or osteogenic medium. Cell surface staining for flow cytometry was performed using anti-human ALP antibody Alexa Fluor 700 (Clone B4-78, R&D Systems) as^7^.

### Photoswitching Dendra2

Photoswitching was optimized in a Leica TCS SP5 II laser scanning confocal microscope (Leica Microsystems) by using the Leica LAS AF software FRAP Wizard application (version 2.6.0.7266, Leica Microsystems) and applying a ROI (region of interest) scan, with a 10x HCX PL APO CS dry objective (0.4 N.A.) with the pinhole set to Airy1 (53.07 μm), image size 1024 × 1024 pixels with 16 bits and bidirectional scanning at 400 Hz. Cells were firstly imaged by sequentially acquiring green and red images with a 488 nm and 561 nm laser lines in low power. Dendra2 was converted to its red form by exposure to 405 nm laser using distinct iteration number (defined as one complete pass with the 405 nm laser, 1.303 s/frame, laser power at 2.466 mW) for a ROI with 470,000 μm^2^. Finally, cells were imaged again in the green and red channels to quantify photoconversion efficiency. For 3D cell tracking 3 to 6 spots were photoswitched for each material in each individual experiment.

### Wound healing assay combined with time-lapse microscopy

MSC were plated at 25,000 cells/well in 24-well plates in MSC growth medium. When cells reached 100% confluence a scratch was made using a 200 μL pipette tip. Cell monolayer was washed twice with PBS, MSC growth medium was added and cells were imaged in a confocal microscope (Leica TCS SP5 II, Leica Microsystems, Wetzlar, Germany), at 37 °C/5% CO_2_ (incubator from OKO lab). Bright-field and fluorescence images were collected using the Live Data Mode application, with 10x dry objective. Images were captured every 15 min for 16–24 h. Each experiment was run in triplicate and repeated 4 times. For each replicate 3 distinct positions were imaged. Photoswitching of transfected MSC located at the edges of the wound was performed immediately after the scratch. Single cell trajectories in time-lapse movies were analyzed using Gradientech Tracking Tool™ v1.07 software (Gradientech). Only cells initially at the migrating front were considered for analysis. The directness index is a measure of the cell directionality and is calculated by comparing euclidean and accumulated distances. This value can range from 0 to 1, with a value closer to 1 indicating oriented cell migration.

### Preparation of 3D matrices

Matrigel: Matrigel Matrix Phenol Red-free (BD Biosciences) was mixed with cells following manufacturer’s instructions and allowed to gel at 37 °C.

RGD-alginate disks: PRONOVA ultrapure sodium alginate (FMC Biopolymers, 70% guluronic acid) was covalently modified with cell-adhesion peptide sequence GGGGRGDSP (abbreviated as RGD, custom-made at GenScript (U.S.A.)) using aqueous carbodiimide chemistry, as previously described^9^. Solutions composed of a 1:1 mixture of high (HMW, 1.5 × 10^5 ^Da) and low molecular weight (LMW, 2.5 × 10^4^ Da) alginate (both RGD-modified) were prepared in 0.9 wt-% sodium chloride (NaCl, Sigma) and combined with crosslinking agents (CaCO_3_ and δ-gluconolactone (GDL)) to trigger hydrogel formation by internal gelation^1^.

Chitosan scaffolds: Chitosan (France-Chitine) was purified as described^2^. Chitosan sponges were prepared by freeze-drying using a 2% solution of chitosan (degree of acetylation: 12.00 ± 2.35%, molecular weight: 324 ± 27 × 10^3^) and disinfected as previously described^3^. Scaffolds were cut in parallelepiped shape with a dimension of 4 × 4 mm and a height of 2 mm (1 ± 0.2 mg average weight).

### MSC seeding in 3D matrices

Matrigel: 1.8 × 10^5^ transfected cells were mixed with Matrigel solution pre-cooled at 4 °C at 5.2 or 9.6 mg/mL of protein content. Solution was cast into a 0.8 cm^2^ Lab-Tek II chamber and incubated at 37 °C/5% CO_2_ for 30 min, until gelling was complete.

RGD-alginate: transfected MSC were homogeneously mixed with gel precursor solutions, prepared as described above, for a final concentration of 8 × 10^6^ cells/mL and 1 or 2% (wt) alginate (both with RGD at 100 μM). Crosslinking of hydrogel disks was promoted in teflon plates by placing 15 μL of the solution into the plate surface. Small and uniform hydrogel disks (1.5 mm height, 3 mm diameter) were obtained by mechanical pressing during the gelling process using 3 mm spacers. Samples were incubated at 37 °C/5% CO_2_ for 1 h, until crosslinking was completed.

Chitosan: 2.5 × 10^5^ cells in 10  μL of MSC growth medium were added to each side (top and bottom) of a scaffold in non-treated 24 well tissue culture plates. Cells were allowed to adhere to the scaffold for 4 h and 400 μL of MSC growth medium was added.

Prior to imaging, chitosan and RGD-alginate matrices were transferred to a 10 cm^2^ dish with a bottom coverslip and to a 0.8 cm^2^ Lab-Tek II chamber, respectively. After 24 h of incubation cells were photoswitched and imaged. The cell-loaded matrices were cultured during seven days and medium was changed every 48 h.

### Tracking Photoswitched MSC

The reference red cell population (3–6 spots per scaffolds in each individual experiment) was centered and imaged 0, 1, 4 and 7 days after conversion. Each spot was imaged by sequentially acquiring green and red images with 3% power of the 488 nm laser and with a 30% power of the 561 nm laser using a 10x dry objective, pinhole set to Airy1, image size 512 × 512 pixels with 8 bits, bidirectional scanning at 400 Hz, using xyz mode with a Z-stack of 50 frames, each with 9.99 μm thickness, building a Z-stack with 499.5 μm. After imaging, cells were returned to the incubator and cultured at 37 °C/5% CO_2_, until the next imaging time-point.

### Data processing

#### Photoconversion efficiency

Fluorescence intensity of both green and red forms of the Dendra2 protein were measured in 16 bits images using ImageJ software. The increase in fluorescence of Green and Red Signal Post-Conversion (relative fluorescence intensity, RFU) was normalized to take into account the initial number of Dendra2 green fluorescence molecules by calculating:


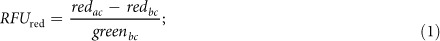



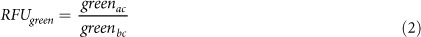


where red_ac _= red fluorescence intensity after conversion; red_bc _= red fluorescence intensity before conversion; green_ac _= green fluorescence intensity after conversion; green_bc _= green fluorescence intensity before conversion.

#### Cell tracking and shape analysis

To quantify the cells infiltration area, 2D maximum projection images of Z-stacks for both green and red channels were generated using Leica LAS AF software for each photoswitched spot. The same manual threshold (threshold value ≥30; scale 0–255) was applied to all images using ImageJ software. The area occupied by red cells was quantified by drawing a manual ROI around this area (Supplementary Fig. 3). The infiltration area was then normalized to the area measured at time-point 0.

For shape analysis, ICY software was used^4^. Images were threshold by the Otsu method with the Best Threshold plugin (developed by Thomas Provoost)^5^. Then, Spot Detector plugin (developed by Fabrice de Chaumont)^6^ was used for detection of the 3D objects followed by 3D Analysis (developed by Thomas Boudier)^7^ to calculate object sphericity.

### Statistical analysis

Statistical analysis was performed using Prism5 software, v5.01 for data on MSC characterization (Fig. 1, 2, 3). One tailed Mann-Whitney test was used to compare two samples, whereas comparison between more than two samples was performed using the Friedman-matched paired test followed by Dunns comparison test. For grouped samples, two-way ANOVA test was used followed by Bonferroni post-test.

R software (v2.14.1) was used for statistical analysis of cells motility and shape (Fig. 4, 5, 6). To test cell velocity, directness index, relative infiltration area and cell sphericity for significant differences between groups we used a linear mixed model^8^, as detailed in Supplementary Methods. For 2D migration experiments, groups were treated as fixed effects and individual cells nested within each experiment were treated as random factors. For infiltration area and cell sphericity, spots and cells, respectively, were considered nested, with days nested within each experiment, which was treated as a random effect to take into account possible variability. The detailed results of this statistical analysis are in Supplementary Tables 1–5.

Data are mean ± Standard Error of the Mean (SEM) unless stated otherwise. Confidence intervals (CIs) for the desired comparisons were computed as^9^.

Parameters were estimated by computing the maximum likelihood estimators using R software (www.R-project.org, R Development Core Team) and the lme and multcomp package. Values of p < 0.05 (^*^), p < 0.01 (^**^) and p < 0.001 (^***^) were considered statistically significant.

## Author Contributions

H.R.C. designed the experiments, acquired and analyzed the data and wrote the manuscript M.G.L. designed the experiments, acquired and analyzed the data C.O. analyzed the data and wrote the manuscript D.G., D.M. acquired the data C.O., C.B., M.A.B. designed the experiments and analyzed the data C.R.A. designed and supervised the experiments, analyzed the data and wrote the manuscript

## Additional Information

**How to cite this article**: Caires, H. R. *et al.* Finding and tracing human MSC in 3D microenvironments with the photoconvertible protein Dendra2. *Sci. Rep.* **5**, 10079; doi: 10.1038/srep10079 (2015).

## Supplementary Material

Supplementary Information

## Figures and Tables

**Table 1:** Comparison of scaffold characteristics, imaging strategies and MSC behaviour in 3D Matrigel, RGD-Alginate and Chitosan microenvironments.

		**Matrigel**		**RGD-Alginate**		**Chitosan**
		**5.2 mg protein/mL**	**9.6 mg protein/mL**	**1% polymer**	**2% polymer**	
**Scaffold characteristics**	Mesh/Pore Size	≈2 μm^6^	<0.5 μm^6^	≈12.1 nm^2^	≈5.3 nm^2^	≈147 μm (large pore) and 58 μm (small pore)^7^
	Stiffness (storage modulus)	≈10 Pa^6^	≈50 Pa^6^	≈99 Pa^2^	≈461 Pa^2^	≈83 KPa^0^
**Cell seeding**		Embedded	Embedded	Embedded	Embedded	On top
**Imaging features**	Depth (μm)	400-500	400-500	400-500	400-500	100
	Laser scattering	Low	Low	Low	Low	High
**Cellular behaviour**	Cell sphericity (over 7 days)	Maintain	Decrease	Decrease	Decrease	Maintain
	% Infiltration area (after 7 days)	259	193	262	207	265

**Table 2:** Key characteristics of using photoconvertible or fluorescent proteins to label and quantify cell motility in 3D matrices over long periods of time

	**Photoconvertible Proteins (e.g. Dendra2)**	**Fluorescent Proteins (e.g. GFP)**
Brightness	19.3*	16.17*
Photostability	+++	+++
Need for continuous live imaging	No. Allows imaging for more than 15 days.	Preferable. Sample should not be moved from the initial position throughout imaging.
Imaging of the same cell(s) over days	Yes. Technically easy to create a reference by photomarking single cells or a defined subpopulation that fluoresce at a specific wavelength.	Need for cell registry strategies that are technically demanding. Small movement of the scaffold will result in misalignment of the tracked cells^6^.
Effect of cell density on 3D tracking	Efficient in a range of cell densities.	Limited to imaging at low cell densities as otherwise it is difficult to follow cell trajectories.

^*^Brightness is calculated as a product of molar extinction coefficient (EC) and fluorescence quantum yield (QY) in mM•cm^−1^
