# Supplementary material for: Finding and tracing human MSC in 3D microenvironments with the photoconvertible protein Dendra2
Source: Sci Rep. 2015 May 14;5:10079. doi: 10.1038/srep10079 (PMC4431349; doi:10.1038/srep10079)
Supplement: Supplementary Information [file srep10079-s1.doc]

# Supplementary information

# Finding and tracing human MSC in 3D microenvironments with the photoconvertible protein Dendra2

Hugo R. Caires, Maria Gomez-Lazaro, Carla M. Oliveira, David Gomes, Denisa D. Mateus, Carla Oliveira, Cristina C. Barrias, Mário A. Barbosa, Catarina R. Almeida

**Supplementary Methods**

## Oil Red O / ALP / Von Kossa Stainings

For adipogenesis or osteogenesis, hMSCs were plated in 24 well plates until reaching confluence and were stimulated with DMEM with low glucose and glutamax plus 10% FBS (PAA) and penicillin/streptomycin and with or without the adipogenic or osteogenic supplements as previously described^1^. Cells were washed with distilled water twice and analyzed both under an inverted microscope (Olympus CKX Inverted Microscope) and in a confocal microscope (Leica TCS SP5 II) for co-localization of Oil Red O stained lipid droplets or ALP with cells Dendra2 fluorescence.

**Statistical analysis: Linear Mixed Model Analysis**

For 2D migration experiments, groups were treated as fixed effects and individual cells nested within each experiment were treated as random factors. For infiltration area and cell sphericity, spots and cells, respectively, were considered nested, with days nested within each experiment, which was treated as a random effect to take into account possible variability. The fixed effect materials and days interaction was tested for significance. The random factor experiment described the between-experiment variability of infiltration area and sphericity from one experiment to another, the days described the between-days-within-experiment variability from one day to another one, and the random factor cells described the between-cells-within-days-within-experiment variability of infiltration area and sphericity. This random effect was assumed to follow a normal distribution centered on 0 with standard deviations for experiment, days and cells.

To assess the importance of random factors in our model, we tested their significance by removing terms one at a days and comparing the difference between the log likelihoods of the reduced and complete models using a qui-square test. In each model random terms were retained if they significantly improved likelihood.

# References

1. Almeida CR, Vasconcelos DP, Goncalves RM, Barbosa MA. Enhanced mesenchymal stromal cell recruitment via natural killer cells by incorporation of inflammatory signals in biomaterials. *J R Soc Interface* **9**, 261-271 (2012).

# Supplementary Figures


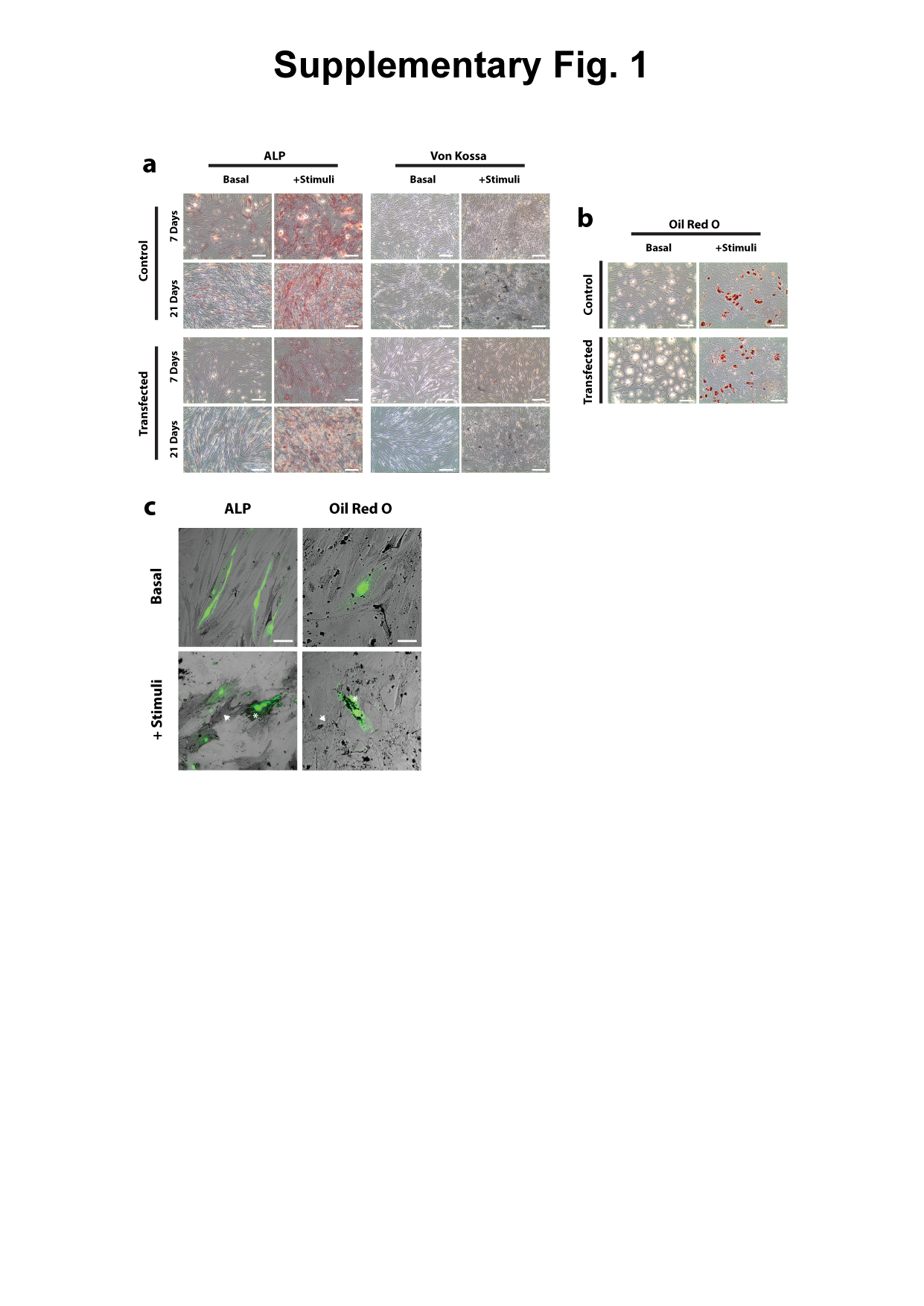


**Supplementary Figure 1. Transfected MSC are capable of multilineage differentiation when appropriately stimulated but not in basal condition.** (a) Control and transfected MSC differentiate into osteoblasts as determined by staining for ALP activity (left: ALP staining) and late calcium-containing mineral deposits in extracellular matrix (right: von Kossa staining). (b) Lipid vacuoles accumulation stained with Oil Red O solution at 21 days indicates differentiating adipocytes on both control and transfected MSC. Scale bar, 250 µm. (c) Both Dendra2+ (asterisk) and Dendra2- MSC (arrowhead) were able to differentiate into osteogenic or adipogenic lineages when appropriately stimulated as denoted by expression of ALP showed as black staining (left) or oil droplets illustrated as black droplets (right), respectively. Scale bar, 75 µm. Pictures are representative of 3 individual experiments in duplicate.

**
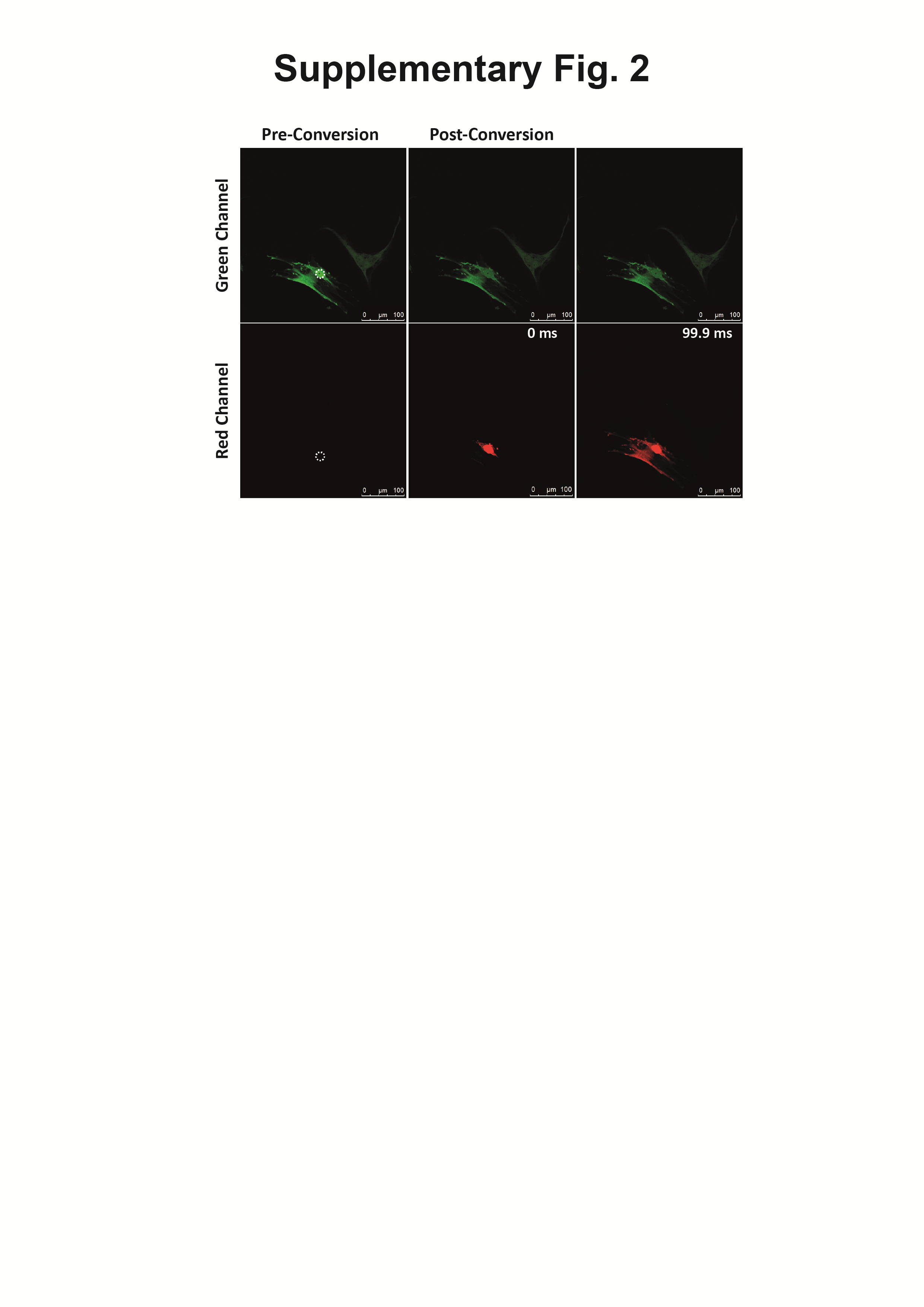
**

**Supplementary Figure 2. Photoconversion at sub-cellular resolution.** Confocal microscopy images in Green (top) and Red (bottom) channels of Dendra2+ MSC. Photoconversion was achieved with 405nm laser in the circular region of Interest indicated (dashed line). Photoswitched red Dendra2 redistributed throughout the cell cytoplasm within 99.9 ms after conversion.


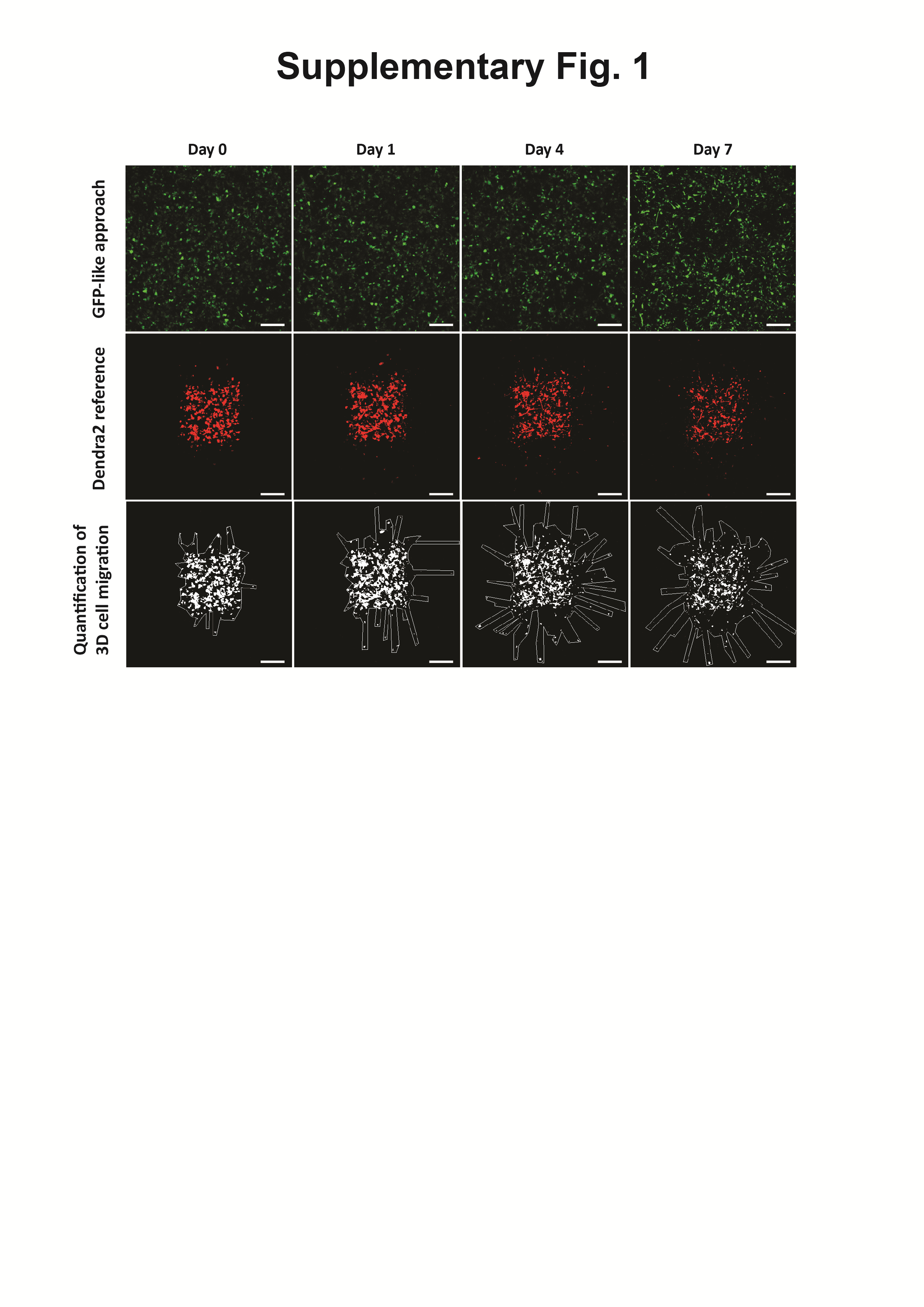


**Supplementary Figure 3. Using Dendra2 photoconversion to track MSC.** (top) It is difficult to quantify cell motility in 3D when MSC are all labeled with the same fluorescent protein (GFP-like approach, cells were imaged in the green channel only). In this scenario, it is extremely hard to find the same cells when imaging sessions have to be performed at discrete daily intervals and samples cannot be kept under a microscope. (middle) Dendra2 photoconversion of a group of cells creates a fixed reference with defined geometric shapes that allows repositioning the same cells over the different imaging sessions. (bottom) Infiltration area quantified in xy maximum projection images by manual drawing a ROI. Scale bar, 250 µm.

Supplementary Table 1 - Contrast comparison using mixed effect model fit to test differences in mean directness index between groups. Group1: Control ; Group2: Electroporated without plasmid ; Group3: Dendra2+ Green ; Group4: Dendra2+ Red.

| **Materials** | **Estimate** | **Std. Error** | **95%CI** | **z value** | **Pr(>|z|)** |  |
| --- | --- | --- | --- | --- | --- | --- |
| Group2 vs. Group1 | -0.017 | 0.015 | (-0.055; 0.020) | -1.179 | 0.637 |  |
| Group3 vs. Group1 | -0.001 | 0.015 | (-0.041; 0.039) | -0.056 | 1.000 |  |
| Group4 vs. Group1 | -0.047 | 0.019 | (-0.096; 0.002) | -2.452 | 0.066 |  |
| Group3 vs. Group2 | 0.017 | 0.014 | (-0.020; 0.053) | 1.176 | 0.639 |  |
| Group4 vs. Group2 | -0.029 | 0.018 | (-0.075; 0.017) | -1.630 | 0.358 |  |
| Group4 vs. Group3 | -0.046 | 0.019 | (-0.094; 0.002) | -2.476 | 0.062 |  |
| **Variance Component** | | |  | **StdDev** |  |  |
| Between-experiment |  |  |  | 0.000002 |  |  |
| Between-cells-within-experiments | |  |  | 0.000009 |  |  |
| Residuals |  |  |  | 0.1335 |  |  |

Supplementary Table 2 - Contrast comparison using mixed effect model fit to test differences in mean velocity between groups. Group1: Control ; Group2: Electroporated without plasmid ; Group3: Dendra2+ Green ; Group4: Dendra2+ Red.

|  | **Estimate** | **Std. Error** | **95%CI** | **z value** | **Pr(>|z|)** |  |
| --- | --- | --- | --- | --- | --- | --- |
| Group2 vs. Group1 | 0.030216 | 0.013872 | (-0.005; 0.066) | 2.178 | 0.128 |  |
| Group3 vs. Group1 | -0.10191 | 0.014061 | (-0.138; -0.066) | -7.248 | p<0.001 | *** |
| Group4 vs. Group1 | -0.11584 | 0.01737 | (-0.160; -0.071) | -6.669 | p<0.001 | *** |
| Group3 vs. Group2 | -0.13212 | 0.013375 | (-0.166; -0.098) | -9.879 | p<0.001 | *** |
| Group4 vs. Group2 | -0.14605 | 0.016651 | (-0.189; -0.103) | -8.772 | p<0.001 | *** |
| Group4 vs. Group3 | -0.01393 | 0.016884 | (-0.057; 0.029) | -0.825 | 0.841 |  |
| Variance Component | | |  | StdDev |  |  |
| between-experiment |  |  |  | 0.0585 |  |  |
| between-cell-within-experiment | |  |  | 0.0043 |  |  |
| Residual |  |  |  | 0.1209 |  |  |
|  |  |  |  |  |  |  |

Supplementary Table 3 - Contrast comparison using mixed effect model fit to test differences in the mean % infiltration area between material in each time point and within material in each time point. Group1: Matrigel 5.2 mg/mL ; Group2: Matrigel 9.6 mg/mL ; Group3: 1% RGD-alginate ; Group4: 2% RGD-alginate ; Group5: Chitosan.

| **Material** | | **Estimate** | | **Std. Error** | | **IC95%** | | **z value** | | **Pr(>|z|)** |  |
| --- | --- | --- | --- | --- | --- | --- | --- | --- | --- | --- | --- |
| TimePoint1: Group2 vs. Group1 | | -22.866 | | 9.959 | | (-50.019; 4.287) | | -2.296 | | 0.146 |  |
| TimePoint1: Group3 vs. Group1 | | -24.884 | | 9.651 | | (-51.195; 1.428) | | -2.578 | | 0.074 |  |
| TimePoint1: Group4 vs. Group1 | | -19.920 | | 9.658 | | (-46.252; 6.413) | | -2.062 | | 0.236 |  |
| TimePoint1: Group5 vs. Group1 | | 29.815 | | 10.629 | | (0.835; 58.795) | | 2.805 | | 0.040 | * |
| TimePoint1: Group3 vs. Group2 | | -2.017 | | 9.651 | | (-28.329; 24.294) | | -0.209 | | 1.000 |  |
| TimePoint1: Group4 vs. Group2 | | 2.946 | | 9.658 | | (-23.386; 29.279) | | 0.305 | | 0.998 |  |
| TimePoint1: Group5 vs. Group2 | | 52.681 | | 10.629 | | (23.701; 81.661) | | 4.956 | | 0.000 | *** |
| TimePoint1: Group4 vs. Group3 | | 4.964 | | 9.330 | | (-20.474; 30.402) | | 0.532 | | 0.984 |  |
| TimePoint1: Group5 vs. Group3 | | 54.699 | | 10.341 | | (26.506; 82.892) | | 5.290 | | 0.000 | *** |
| TimePoint1: Group5 vs. Group4 | | 49.735 | | 10.341 | | (21.540; 77.930) | | 4.809 | | 0.000 | *** |
| TimePoint4: Group2 vs. Group1 | | -37.789 | | 9.959 | | (-64.938; -10.64) | | -3.794 | | 0.001 | ** |
| TimePoint4: Group3 vs. Group1 | | -9.145 | | 9.651 | | (-35.454; 17.163) | | -0.948 | | 0.878 |  |
| TimePoint4: Group4 vs. Group1 | | -9.075 | | 9.658 | | (-35.404; 17.254) | | -0.940 | | 0.881 |  |
| TimePoint4: Group5 vs. Group1 | | 32.153 | | 10.629 | | (3.178; 61.129) | | 3.025 | | 0.021 | * |
| TimePoint4: Group3 vs. Group2 | | 28.644 | | 9.651 | | (2.336; 54.952) | | 2.968 | | 0.025 | * |
| TimePoint4: Group4 vs. Group2 | | 28.715 | | 9.658 | | (2.386; 55.044) | | 2.973 | | 0.024 | * |
| TimePoint4: Group5 vs. Group2 | | 69.943 | | 10.629 | | (40.967; 98.918) | | 6.580 | | 0.000 | *** |
| TimePoint4: Group4 vs. Group3 | | 0.071 | | 9.330 | | (-25.364; 25.506) | | 0.008 | | 1.000 |  |
| TimePoint4: Group5 vs. Group3 | | 41.299 | | 10.341 | | (13.110; 69.488) | | 3.994 | | 0.001 | *** |
| TimePoint4: Group5 vs. Group4 | | 41.228 | | 10.341 | | (13.037; 69.419) | | 3.987 | | 0.001 | *** |
| TimePoint7: Group2 vs. Group1 | | -64.232 | | 9.959 | | (-91.389; -37.074) | | -6.450 | | 0.000 | *** |
| TimePoint7: Group3 vs. Group1 | | 3.796 | | 9.651 | | (-22.520; 30.113) | | 0.393 | | 0.995 |  |
| TimePoint7: Group4 vs. Group1 | | -47.273 | | 9.658 | | (-73.610; -20.935) | | -4.895 | | 0.000 | *** |
| TimePoint7: Group5 vs. Group1 | | 6.079 | | 10.629 | | (-22.906; 35.063) | | 0.572 | | 0.979 |  |
| TimePoint7: Group3 vs. Group2 | | 68.028 | | 9.651 | | (41.711; 94.344) | | 7.049 | | 0.000 | *** |
| TimePoint7: Group4 vs. Group2 | | 16.959 | | 9.658 | | (-9.378; 43.297) | | 1.756 | | 0.399 |  |
| TimePoint7: Group5 vs. Group2 | | 70.310 | | 10.629 | | (41.326; 99.295) | | 6.615 | | 0.000 | *** |
| TimePoint7: Group4 vs. Group3 | | -51.069 | | 9.330 | | (-76.512; -25.626) | | -5.474 | | 0.000 | *** |
| TimePoint7: Group5 vs. Group3 | | 2.282 | | 10.341 | | (-25.916; 30.480) | | 0.221 | | 0.999 |  |
| TimePoint7: Group5 vs. Group4 | | 53.351 | | 10.341 | | (25.151; 81.551) | | 5.159 | | 0.000 | *** |
| **Material** | **Estimate** | | **Std. Error** | | **IC95%** | | **z value** | | **Pr(>|z|)** | |  |
| Group1: Time1 vs. Time0 | 35.634 | | 9.959 | | (10.054; 61.213) | | 3.578 | | 0.002 | | ** |
| Group1: Time4 vs. Time0 | 87.683 | | 9.959 | | (62.103; 113.262) | | 8.804 | | 0.000 | | *** |
| Group1: Time7 vs. Time0 | 160.315 | | 9.959 | | (134.736; 185.895) | | 16.097 | | 0.000 | | *** |
| Group1: Time4 vs. Time1 | 52.049 | | 9.959 | | (26.470; 77.628) | | 5.226 | | 0.000 | | *** |
| Group1: Time7 vs. Time1 | 124.682 | | 9.959 | | (99.102; 150.261) | | 12.519 | | 0.000 | | *** |
| Group1: Time7 vs. Time4 | 72.633 | | 9.959 | | (47.053; 98.212) | | 7.293 | | 0.000 | | *** |
| Group2: Time1 vs. Time0 | 12.767 | | 9.959 | | (-12.741; 38.276) | | 1.282 | | 0.601 | |  |
| Group2: Time4 vs. Time0 | 64.817 | | 14.084 | | (28.742; 100.891) | | 4.602 | | 0.000 | | *** |
| Group2: Time7 vs. Time0 | 137.449 | | 14.084 | | (101.375; 173.523) | | 9.759 | | 0.000 | | *** |
| Group2: Time4 vs. Time1 | 37.126 | | 9.959 | | (11.617; 62.634) | | 3.728 | | 0.001 | | ** |
| Group2: Time7 vs. Time1 | 83.316 | | 9.959 | | (57.808; 108.824) | | 8.366 | | 0.000 | | *** |
| Group2: Time7 vs. Time4 | 46.190 | | 9.959 | | (20.682; 71.699) | | 4.638 | | 0.000 | | *** |
| Group3: Time1 vs. Time0 | 10.627 | | 9.316 | | (-13.649; 34.903) | | 1.141 | | 0.768 | |  |
| Group3: Time4 vs. Time0 | 67.472 | | 13.637 | | (31.935; 103.008) | | 4.948 | | 0.000 | | *** |
| Group3: Time7 vs. Time0 | 189.605 | | 15.014 | | (150.481; 228.729) | | 12.629 | | 0.000 | | *** |
| Group3: Time4 vs. Time1 | 67.787 | | 9.316 | | (43.511; 92.063) | | 7.277 | | 0.000 | | *** |
| Group3: Time7 vs. Time1 | 153.362 | | 9.316 | | (129.085; 177.638) | | 16.462 | | 0.000 | | *** |
| Group3: Time7 vs. Time4 | 85.574 | | 9.316 | | (61.298; 109.850) | | 9.186 | | 0.000 | | *** |
| Group4: Time1 vs. Time0 | 15.422 | | 9.316 | | (-8.475; 39.320) | | 1.656 | | 0.359 | |  |
| Group4: Time4 vs. Time0 | 67.472 | | 13.637 | | (32.489; 102.455) | | 4.948 | | 0.000 | | *** |
| Group4: Time7 vs. Time0 | 140.104 | | 13.637 | | (105.121; 175.087) | | 10.274 | | 0.000 | | *** |
| Group4: Time4 vs. Time1 | 62.894 | | 9.316 | | (38.996; 86.792) | | 6.751 | | 0.000 | | *** |
| Group4: Time7 vs. Time1 | 97.329 | | 9.316 | | (73.431; 121.227) | | 10.448 | | 0.000 | | *** |
| Group4: Time7 vs. Time4 | 34.435 | | 9.316 | | (10.537; 58.333) | | 3.696 | | 0.001 | | ** |
| Group5: Time1 vs. Time0 | 64.923 | | 11.235 | | (36.216; 93.631) | | 5.778 | | 0.000 | | *** |
| Group5: Time4 vs. Time0 | 116.972 | | 15.014 | | (78.610; 155.335) | | 7.791 | | 0.000 | | *** |
| Group5: Time7 vs. Time0 | 189.605 | | 15.014 | | (151.243; 227.967) | | 12.629 | | 0.000 | | *** |
| Group5: Time4 vs. Time1 | 54.387 | | 11.235 | | (25.680; 83.095) | | 4.841 | | 0.000 | | *** |
| Group5: Time7 vs. Time1 | 100.945 | | 11.235 | | (72.238; 129.653) | | 8.985 | | 0.000 | | *** |
| Group5: Time7 vs. Time4 | 46.558 | | 11.235 | | (17.850; 75.265) | | 4.144 | | 0.000 | | *** |
| **Variance Component** |  | |  | |  | | **StdDev** | |  | |  |
| between-experiment | | | | |  | | 0.0027 | |  | |  |
| between-spot-within-experiment | | | | |  | | 4.4 | |  | |  |
| between-days-within-spot-within-experiment | | | | |  | | 0.0002 | |  | |  |
| Residual | | | | |  | | 26.349 | |  | |  |

**Supplementary Table 4 - Contrast comparison using mixed effect model fit to test differences in cell sphericity between material in each time point and within material over time. Group1: Matrigel 5.2 mg/mL ; Group2: Matrigel 9.6 mg/mL ; Group3: 1% RGD-alginate ; Group4: 2% RGD-alginate ; Group5: Chitosan.**

| **Material** | **Estimate** | **Std. Error** | **IC95%** | **z value** | **Pr(>|z|)** |  |
| --- | --- | --- | --- | --- | --- | --- |
| TimePoint0: Group2 vs. Group1 | 0.046 | 0.007 | (0.027; 0.065) | 6.500 | 0.000 | *** |
| TimePoint0: Group3 vs. Group1 | 0.007 | 0.007 | (-0.011; 0.025) | 1.085 | 0.814 |  |
| TimePoint0: Group4 vs. Group1 | 0.016 | 0.007 | (-0.003; 0.035) | 2.263 | 0.157 |  |
| TimePoint0: Group5 vs. Group1 | -0.069 | 0.007 | (-0.09; -0.049) | -9.319 | 0.000 | *** |
| TimePoint0: Group3 vs. Group2 | -0.039 | 0.006 | (-0.056; -0.021) | -5.936 | 0.000 | *** |
| TimePoint0: Group4 vs. Group2 | -0.030 | 0.007 | (-0.049; -0.011) | -4.275 | 0.000 | *** |
| TimePoint0: Group5 vs. Group2 | -0.115 | 0.007 | (-0.135; -0.095) | -15.658 | 0.000 | *** |
| TimePoint0: Group4 vs. Group3 | 0.009 | 0.006 | (-0.009; 0.026) | 1.367 | 0.648 |  |
| TimePoint0: Group5 vs. Group3 | -0.077 | 0.007 | (-0.095; -0.058) | -11.131 | 0.000 | *** |
| TimePoint0: Group5 vs. Group4 | -0.085 | 0.007 | (-0.105; -0.066) | -11.698 | 0.000 | *** |
| TimePoint1: Group2 vs. Group1 | 0.029 | 0.007 | (0.029; 0.011) | 4.269 | 0.000 | *** |
| TimePoint1: Group3 vs. Group1 | 0.005 | 0.007 | (0.005; -0.013) | 0.772 | 0.939 |  |
| TimePoint1: Group5 vs. Group1 | -0.087 | 0.007 | (-0.087; -0.106) | -12.157 | 0.000 | *** |
| TimePoint1: Group4 vs. Group1 | 0.019 | 0.007 | (0.019; 0.000) | 2.682 | 0.056 |  |
| TimePoint1: Group3 vs. Group2 | -0.024 | 0.007 | (-0.024; -0.042) | -3.62 | 0.003 | ** |
| TimePoint1: Group4 vs. Group2 | -0.011 | 0.007 | (-0.011; -0.029) | -1.546 | 0.532 |  |
| TimePoint1: Group5 vs. Group2 | -0.116 | 0.007 | (-0.116; -0.135) | -16.375 | 0.000 | *** |
| TimePoint1: Group4 vs. Group3 | 0.013 | 0.007 | (0.013; -0.005) | 2.016 | 0.258 |  |
| TimePoint1: Group5 vs. Group3 | -0.092 | 0.007 | (-0.092; -0.111) | -13.43 | 0.000 | *** |
| TimePoint1: Group5 vs. Group4 | -0.106 | 0.007 | (-0.106; -0.125) | -14.971 | 0.000 | *** |
| TimePoint4: Group2 vs. Group1 | 0.016 | 0.006 | (0.016; 0.000) | 2.777 | 0.043 | * |
| TimePoint4: Group3 vs. Group1 | -0.027 | 0.005 | (-0.027; -0.042) | -4.983 | 0.000 | *** |
| TimePoint4: Group4 vs. Group1 | -0.021 | 0.006 | (-0.021; -0.037) | -3.721 | 0.002 | ** |
| TimePoint4: Group5 vs. Group1 | -0.078 | 0.007 | (-0.078; -0.096) | -11.946 | 0.000 | *** |
| TimePoint4: Group3 vs. Group2 | -0.043 | 0.005 | (-0.043; -0.057) | -8.017 | 0.000 | *** |
| TimePoint4: Group4 vs. Group2 | -0.037 | 0.006 | (-0.037; -0.053) | -6.58 | 0.000 | *** |
| TimePoint4: Group5 vs. Group2 | -0.094 | 0.006 | (-0.094; -0.111) | -14.575 | 0.000 | *** |
| TimePoint4: Group4 vs. Group3 | 0.006 | 0.005 | (0.006; -0.009) | 1.053 | 0.829 |  |
| TimePoint4: Group5 vs. Group3 | -0.051 | 0.006 | (-0.051; -0.068) | -8.258 | 0.000 | *** |
| TimePoint4: Group5 vs. Group4 | -0.057 | 0.006 | (-0.057; -0.074) | -8.771 | 0.000 | *** |
| TimePoint7: Group2 vs. Group1 | 0.046 | 0.007 | (0.046; 0.026) | 6.5 | 0.000 | *** |
| TimePoint7: Group3 vs. Group1 | 0.007 | 0.007 | (0.007; -0.011) | 1.085 | 0.892 |  |
| TimePoint7: Group4 vs. Group1 | 0.016 | 0.007 | (0.016; -0.003) | 2.263 | 0.175 |  |
| TimePoint7: Group5 vs. Group1 | -0.069 | 0.007 | (-0.069; -0.090) | -9.319 | 0.000 | *** |
| TimePoint7: Group3 vs. Group2 | -0.046 | 0.007 | (-0.046; -0.066) | -6.51 | 0.000 | *** |
| TimePoint7: Group4 vs. Group2 | -0.027 | 0.008 | (-0.027; -0.048) | -3.525 | 0.004 | ** |
| TimePoint7: Group5 vs. Group2 | -0.093 | 0.007 | (-0.093; -0.113) | -12.811 | 0.000 | *** |
| TimePoint7: Group4 vs. Group3 | 0.020 | 0.006 | (0.020; 0.002) | 3.131 | 0.016 | * |
| TimePoint7: Group5 vs. Group3 | -0.047 | 0.006 | (-0.047; -0.064) | -7.671 | 0.000 | *** |
| TimePoint7: Group5 vs. Group4 | -0.066 | 0.007 | (-0.066; -0.084) | -10.099 | 0.000 | *** |
| **Material** | **Estimate** | **Std. Error** | **IC95%** | **z value** | **Pr(>|z|)** |  |
| Group1: Time1 vs. Time0 | 0.007 | 0.009 | (0.007; -0.017) | 0.784 | 0.862 |  |
| Group1: Time4 vs. Time0 | 0.007 | 0.009 | (0.007; -0.016) | 0.817 | 0.846 |  |
| Group1: Time7 vs. Time0 | -0.012 | 0.009 | (-0.012; -0.036) | -1.282 | 0.574 |  |
| Group1: Time4 vs. Time1 | 0.000 | 0.009 | (0.000; -0.023) | -0.001 | 1.000 |  |
| Group1: Time7 vs. Time1 | -0.019 | 0.009 | (-0.019; -0.043) | -2.090 | 0.156 |  |
| Group1: Time7 vs. Time4 | -0.019 | 0.009 | (-0.019; -0.042) | -2.182 | 0.128 |  |
| Group2: Time1 vs. Time0 | -0.009 | 0.009 | (-0.009; -0.033) | -1.000 | 0.797 |  |
| Group2: Time4 vs. Time0 | -0.009 | 0.011 | (-0.009; -0.038) | -0.825 | 0.889 |  |
| Group2: Time7 vs. Time0 | -0.028 | 0.011 | (-0.028; -0.058) | -2.476 | 0.065 |  |
| Group2: Time4 vs. Time1 | -0.013 | 0.009 | (-0.013; -0.036) | -1.520 | 0.451 |  |
| Group2: Time7 vs. Time1 | -0.025 | 0.010 | (-0.025; -0.050) | -2.610 | 0.045 | * |
| Group2: Time7 vs. Time4 | -0.012 | 0.009 | (-0.012; -0.036) | -1.299 | 0.601 |  |
| Group3: Time1 vs. Time0 | 0.005 | 0.009 | (0.005; -0.017) | 0.608 | 0.976 |  |
| Group3: Time4 vs. Time0 | 0.010 | 0.011 | (0.010; -0.019) | 0.878 | 0.896 |  |
| Group3: Time7 vs. Time0 | -0.029 | 0.012 | (-0.029; -0.060) | -2.481 | 0.069 |  |
| Group3: Time4 vs. Time1 | -0.032 | 0.008 | (-0.032; -0.054) | -3.837 | 0.001 | *** |
| Group3: Time7 vs. Time1 | -0.048 | 0.009 | (-0.048; -0.070) | -5.608 | 0.000 | *** |
| Group3: Time7 vs. Time4 | -0.016 | 0.008 | (-0.016; -0.037) | -1.961 | 0.230 |  |
| Group4: Time1 vs. Time0 | 0.010 | 0.009 | (0.010; -0.014) | 1.073 | 0.751 |  |
| Group4: Time4 vs. Time0 | 0.010 | 0.011 | (0.010; -0.019) | 0.878 | 0.863 |  |
| Group4: Time7 vs. Time0 | -0.009 | 0.012 | (-0.009; -0.039) | -0.817 | 0.892 |  |
| Group4: Time4 vs. Time1 | -0.040 | 0.009 | (-0.040; -0.062) | -4.573 | 0.000 | *** |
| Group4: Time7 vs. Time1 | -0.042 | 0.009 | (-0.042; -0.065) | -4.544 | 0.000 | *** |
| Group4: Time7 vs. Time4 | -0.002 | 0.009 | (-0.002; -0.024) | -0.200 | 1.000 |  |
| Group5: Time1 vs. Time0 | -0.010 | 0.010 | (-0.010; -0.035) | -1.067 | 0.752 |  |
| Group5: Time4 vs. Time0 | -0.010 | 0.012 | (-0.010; -0.040) | -0.884 | 0.858 |  |
| Group5: Time7 vs. Time0 | -0.029 | 0.012 | (-0.029; -0.060) | -2.481 | 0.063 |  |
| Group5: Time4 vs. Time1 | 0.009 | 0.009 | (0.009; -0.015) | 0.968 | 0.812 |  |
| Group5: Time7 vs. Time1 | -0.002 | 0.009 | (-0.002; -0.026) | -0.266 | 0.999 |  |
| Group5: Time7 vs. Time4 | -0.011 | 0.009 | (-0.011; -0.035) | -1.274 | 0.614 |  |
| Variance Component | | | | StdDev |  |  |
| between-experiment | | | | 0.005 |  |  |
| between-days-within-experiment | | | | 0.007 |  |  |
| between-cells-within-days-within-experiment | | | | 0.000002 |  |  |
| Residual | | | | 0.0796 |  |  |

Supplementary Table 5 - Contrast comparison using mixed effect model fit to test differences in mean cell sphericity within material over time (consider time continuous).

| **Trend per Material** | **Estimate** | **Std. Error** | **IC95%** | **z value** | **Pr(>|z|)** |  |
| --- | --- | --- | --- | --- | --- | --- |
| Trend in Matrigel 5.2 mg/mL | -0.002 | 0.001 | (-0.005; 0.001) | -1.486 | 0.459 |  |
| Trend in Matrigel 9.6 mg/mL | -0.005 | 0.001 | (-0.008; -0.002) | -3.929 | 0.000 | *** |
| Trend in 1% RGD-alginate | -0.007 | 0.001 | (-0.010; -0.004) | -6.613 | 0.000 | *** |
| Trend in 2% RGD-alginate | -0.006 | 0.001 | (-0.009; -0.003) | -5.288 | 0.000 | *** |
| Trend in Chitosan | -0.001 | 0.001 | (-0.004; 0.002) | -0.935 | 0.840 |  |
|  |  |  |  |  |  |  |
